# *EGFR* and *KRAS* Mutations Predict the Incidence and Outcome of Brain Metastases in Non-Small Cell Lung Cancer

**DOI:** 10.3390/ijms17122132

**Published:** 2016-12-18

**Authors:** Pascale Tomasini, Cindy Serdjebi, Nataliya Khobta, Philippe Metellus, L’Houcine Ouafik, Isabelle Nanni, Laurent Greillier, Anderson Loundou, Frederic Fina, Celine Mascaux, Fabrice Barlesi

**Affiliations:** 1 Assistance Publique Hôpitaux de Marseille, Multidisciplinary Oncology & Therapeutic Innovations department. Aix Marseille University, Marseille 13015, France; cindy.serdjebi@univ-amu.fr (C.S.); nataliyasantoni@hotmail.com (N.K.); laurent.greillier@ap-hm.fr (L.G.); celine.mascaux@ap-hm.fr (C.M.); fabrice.barlesi@ap-hm.fr (F.B.); 2 Inserm U911 CRO2 (Centre de Recherche en Oncologie biologique et Onco-pharmacologie), Aix Marseille University, Marseille 13005, France; L'houcine.OUAFIK@ap-hm.fr; 3 Assistance Publique Hôpitaux de Marseille, Transfer Oncology Laboratory, Aix Marseille University, Marseille 13015, France; Isabelle.NANNI@ap-hm.fr (I.N.); frederic.fina@ap-hm.fr (F.F.); 4 Department of Neurosurgery, Aix-Marseille University, Marseille 13005, France; philippe.metellus@outlook.fr; 5 Statistics Department, Aix Marseille University, Marseille 13005, France; anderson.loundou@univ-amu.fr

**Keywords:** brain metastasis, lung neoplasm, KRAS, EGFR, incidence, recurrence, survival

## Abstract

Background: Lung cancer is the leading cause of brain metastases (BM). The identification of driver oncogenes and matched targeted therapies has improved outcome in non-small cell lung cancer (NSCLC) patients; however, a better understanding of BM molecular biology is needed to further drive the process in this field. Methods: In this observational study, stage IV NSCLC patients tested for *EGFR* and *KRAS* mutations were selected, and BM incidence, recurrence and patients’ outcome were assessed. Results: A total of 144 patients (142 Caucasian and two Asian) were selected, including 11.27% with *EGFR*-mutant and 33.10% with *KRAS*-mutant tumors, and 57.04% patients had developed BM. BM incidence was more frequent in patients with *EGFR* mutation according to multivariate analyses (MVA) (Odds ratio OR = 8.745 [1.743–43.881], *p* = 0.008). Among patients with treated BM, recurrence after local treatment was less frequent in patients with *KRAS* mutation (OR = 0.234 [0.078–0.699], *p* = 0.009). Among patients with untreated BM, overall survival (OS) was shorter for patients with *KRAS* mutation according to univariate analysis (OR = 7.130 [1.240–41.012], *p* = 0.028), but not MVA. Conclusions: *EGFR* and *KRAS* mutations have a predictive role on BM incidence, recurrence and outcome in Caucasian NSCLC patients. These results may impact the routine management of disease in these patients. Further studies are required to assess the influence of other biomarkers on NSCLC BM.

## 1. Introduction

Lung cancer remains the leading cause of cancer-related deaths worldwide [1]. It is also the leading cause of brain metastases (BM), accounting for 40% to 50% of all BM [2]. In autopsy series, BM were found in approximately 50% lung cancer patients [3]. Moreover, BM are described as the primary or contributing cause of death in 50% of patients with BM from solid tumors [4]. Without any treatment, the median overall survival (OS) of lung cancer patients with BM is four to 11 weeks [4]. Local treatments such as neurosurgery, stereotactic radiosurgery (SRS) or whole brain radiation therapy (WBRT) improve local control and are associated with increased median OS of up to 14 months [5]. Furthermore, the combination of antiangiogenic therapies, such as bevacizumab, with chemotherapy increases the OS of patients with NSCLC BM. However, median OS does not exceed 16 months [6].

Over the past decade, advances have been made in the understanding of cancer biology [7]. *EGFR* mutations and *ALK* rearrangements are approved predictive biomarkers for advanced NSCLC targeted treatment. Advanced NSCLC with these genomic alterations can be targeted by tyrosine kinase inhibitors (TKI), including erlotinib [8], gefitinib [9], afatinib [10] or crizotinib [11] as a first line treatment. These therapies improved overall response rates (ORR), progression-free survival (PFS) and OS. Some of these therapies even demonstrated intra-cranial activity in retrospective sub-group analysis of large randomized phase III trials. Intracranial disease control rate (DCR) was 56% (95%, CI 46%–66%) with crizotinib [12] and PFS was improved with afatinib relative to chemotherapy in patients with BM (8.2 versus 5.4 months, respectively; Hazard Ratio (HR) = 0.50; *p* = 0.0297) [13]. More recently, a phase II study of ceritinib in previously treated patients with *ALK*-rearranged NSCLC found an intracranial ORR of 45.0% (95% CI, 23.1% to 68.5%) among the 100 patients with baseline BM [14].

However, tumor genetic alterations are heterogeneous. Brain is considered to be a sanctuary site because of the blood–brain barrier (BBB). The BBB is a physiological obstruction to the delivery of systemic therapies to the brain parenchyma and central nervous system (CNS) [15] and it plays a key role in tumor cell migration and colonization to the brain [16]. For these reasons, there are large differences in biomarkers expressed between the primary tumor and metastases, especially for BM [17]. Overall, lung cancer BM biology is still poorly understood.

To develop new treatments for NSCLC patients with BM, an understanding of BM molecular biology is required. Driver mutations, growth factors and signaling pathways all seem to contribute to BM development [18]. Among lung cancer biomarkers, *EGFR* and *KRAS* are the most frequently mutated genes in lung cancer patients and have been routinely used as biomarkers for a decade.

We therefore conducted an observational study in an attempt to assess the role of *KRAS* and *EGFR* mutations in predicting BM incidence, recurrence as well as survival of NSCLC patients with BM.

## 2. Results

### 2.1. Patient and Tumor Characteristics

Data from a total of 144 patients tested for *EGFR* and *KRAS* mutations between January 2009 and December 2010 at MOTI were obtained. Among these patients, two were excluded because of mutations on both *EGFR* and *KRAS* genes. Out of the 142 remaining patients (48 females and 94 males), 140 (98.59%) were Caucasian and two were Asian (1.41%). Patient and disease characteristics are summarized in Table 1. With respect to metastatic status, 90 patients (63.38%) had only one metastasis, 81 patients (57.04%) had BM and 64.2% of BM patients had synchronous BM.

Sixteen tumor samples (11.27%) were positive for *EGFR* mutations (seven different types of *EGFR* mutation were found: one in exon 18, four in exon 19 and two in exon 21) and 47 (33.10%) tumor samples were positive for *KRAS* mutations (seven different types of *KRAS* mutations were found: five in codon 12 and two in codon 13). Of note, one tumor had two *KRAS* mutations (c.35G>A Gly12Asp; c.38G>A Gly13Asp). The most frequent *EGFR* mutation was the Glu746_Ala750del exon 19 deletion (50.00%), whereas Gly12Cys and Gly12Asp were the most frequent *KRAS* mutations (72.34%). All mutations are described in Table S1.

A total of 125 patients (88.03%) received a systemic treatment, of which a large proportion received chemotherapy (80.28%) relative to EGFR-tyrosine-kinase inhibitors (*n* = 11, 7.75%). Among the 81 patients with BM, 54.32% underwent surgery or stereotactic radiosurgery, while 21 patients (25.93%) received whole brain radiation therapy (WBRT) only. The remaining patients did not receive any BM local treatment. Forty-one patients (50.62%) had a BM recurrence after local treatment.

### 2.2. Association between Mutation Status and Brain Metastases (BM) Incidence and Recurrence

BM incidence was more frequent in patients with *EGFR*-mutant (87.50%) than in patients with *KRAS*-mutant (55.32%) or double WT tumors (51.90%, *p* = 0.031, Table 2). While no difference was seen in BM incidence according to age (*p* = 0.799), sex (*p* = 0.621), ECOG PS (*p* = 0.936), smoking status (*p* = 0.515) or primary tumor local treatment (*p* = 0.498), a higher frequency of BM was observed in patients with *EGFR*-mutant compared to *KRAS*-mutant and double WT tumors in univariate analyses (Odds Ratio OR = 6.488, 95% confidence interval CI [1.383–30.443], *p* = 0.018) (Table 3) and confirmed in multivariate analysis (OR = 8.745, 95%CI [1.743–43.881], *p* = 0.008) (Table 3).

BM recurrence after any local treatment was less frequent in patients with *KRAS*-mutant (30.77%) than in patients with *EGFR*-mutant (57.14%) or in double WT tumors (60.98%, *p* = 0.047, Table 2). While no difference was seen in BM recurrence according to age (*p* = 0.562), sex (*p* = 0.939), ECOG PS (*p* = 0.315), smoking status (*p* = 0.911) or BM local treatment (*p* = 0.709) (Table 3), the lower frequency of BM recurrence was confirmed in patients with *KRAS*-mutant compared to *EGFR*-mutant and double WT tumors in univariate analyses (OR = 0.284, 95%CI [0.100–0.807], *p* = 0.018) (Table 3) and confirmed in multivariate analysis (OR = 0.234, 95%CI [0.078–0.699], *p* = 0.009) (Table 3).

### 2.3. Association between Mutations Status, BM and Survival

In the overall population, mutation status was not associated with OS: OS was 22, 9 and 18 months respectively for patients with *EGFR-*mutant, *KRAS*-mutant or double WT tumors, respectively (*p* = 0.196) (Table 4).

In the subgroup of patients with treated BM, mutation status had no impact on PFS (*p* = 0.227 for surgery and/or SRS; *p* = 0.272 for WBRT only) and OS (*p* = 0.822 for surgery and/or SRS; *p* = 0.208 for WBRT only) (Table 4).

In the subgroup of patients with untreated BM, mutation status was not associated with PFS (PFS for *EGFR*-mutant, *KRAS*-mutant or double WT tumors was 10, 5 and 6 months, respectively, *p* = 0.229). However, OS was significantly less in patients with *KRAS*-mutant (5 months) in comparison with patients with *EGFR*-mutant (19 months) and double WT tumors (14 months, *p* = 0.010) (Table 4). In addition, the number of metastases (*p* = 0.003) and ECOG PS (*p* = 0.011) were significantly associated with OS in univariate analysis (*p* = 0.010) (Table 5). In this subgroup of patients with untreated BM, multivariate analyses did not confirm the association between *KRAS* mutation and OS (OR = 0.356, 95%CI [0.015–8.224], *p* = 0.519) (Table 5).

## 3. Discussion

We analyzed the association of *KRAS* and *EGFR* mutations with brain metastases incidence, recurrence and prognosis in a population of patients with metastatic NSCLC. We observed a higher incidence of BM in patients with *EGFR*-mutant tumors in comparison with *KRAS*-mutant and wild type tumors, independent of systemic treatment or primary lung tumor local treatment. Additionally, although *KRAS*-mutant tumors are considered to be more aggressive tumors [19], we found a lower rate of BM recurrence after local treatment for patients with *KRAS*-mutant relative to *EGFR*-mutant and wild-type tumors. Despite the lower BM incidence and the better control of BM after local treatment in the *KRAS*-mutant group, these patients had a shorter overall survival. This suggests that the poor prognosis of *KRAS*-mutant NSCLC does not rely on BM. Conversely, despite having a higher incidence of BM, patients with *EGFR*-mutant NSCLC have a better prognosis.

The population studied was a homogeneous population of stage IV non-squamous NSCLC patients, representative of other European cohorts, with a very large majority of Caucasian patients. The frequency of *KRAS*-mutant (34%) and *EGFR*-mutant (12.5%) tumors was indeed similar that observed in other Caucasian populations of advanced NSCLC patients [20,21]. Furthermore, we analyzed data not only from patients enrolled in clinical trials, but also from all patients diagnosed in routine practice. For this reason, the population studied here is more representative of NSCLC patient than other studies where stringent exclusion criteria were applied.

Because of the high incidence (approximately 50% of advanced lung cancer patients [3]) and contribution to mortality [4], of BM, a better characterization of the molecular profile associated with BM incidence and prognosis in NSCLC patients was needed. While a positive correlation of *EGFR* mutations with BM is commonly admitted, this is, to our knowledge, the first study to report it in a population of Caucasian patients with advanced NSCLC. These clinically relevant results suggest that patients with *EGFR*-mutant tumors should be carefully monitored for early detection of BM development.

Several case series, reported in Table 6, assess the association of molecular profile with NSCLC BM. However, these series predicted conflicting results due to disparities in the specific types of lung cancer studied, including both squamous and non-squamous and both advanced and early-stage NSCLC. Shin et al. [22] and Guan et al. [23] revealed a positive correlation between *EGFR* mutations and BM. More recently, Baek et al. [24] reported that BM were more frequent in NSCLC patients with *EGFR*-mutant (27.4%) compared to *EGFR* wild type tumors (14.5%, *p* < 0.009) in a cohort of 259 patients with advanced NSCLC. However, these studies were performed in Asian populations. One report demonstrated a positive correlation between *EGFR* mutations and BM in a Caucasian population but it was performed in patients with resected early-stage lung cancer and metastatic recurrence [25].

Other studies found no correlation between BM and molecular profile. For example, Hendricks et al. [26] noted no difference in BM incidence between NSCLC patients with *EGFR*-mutant, *KRAS*-mutant or double wild-type tumors (*p* = 0.645). This is likely due to the design of this controlled study: they enrolled the same number of patients in the three groups, whereas we enrolled all patients diagnosed with advanced NSCLC who underwent prospective molecular testing. Therefore, our study is more representative of stage IV NSCLC patients. In the same way, Li et al. [27] found no relationship between mutation status and BM incidence; however, in this study, biomarkers were analyzed from patient serum instead of tissue, which is a much less sensitive technique. Another study of 100 Asian patients with *EGFR* mutations and BM did not find any difference in the general population but identified a significantly higher incidence of BM for patients with *EGFR* exon 19 deletions (*p* = 0.007) [28]. This positive correlation between *EGFR* exon 19 deletion and BM has been previously reported [29] but we did not find any difference of BM incidence according to the *EGFR* mutation subtype in our study (*p* = 0.072).

Regarding the correlation between mutation profile and BM prognosis, Shin et al. demonstrated that patients with *EGFR*-mutant NSCLC underwent salvage therapy after stereotactic radiosurgery more often than patients with *EGFR* wild-type tumors (*p* = 0.04) [30]. Other studies found molecular profile to be an independent prognostic factor for patients with NSCLC BM. Li et al. noted that *EGFR* exon 19 deletions were associated with a better prognosis (*p* = 0.034) in a population of 106 patients with BM from NSCLC [31]. These results are consistent with ours and suggest that *EGFR* mutations remain a good prognostic factor even if they are associated with a higher rate of BM relapse after local treatment.

Finally, whereas EGFR was proven to be involved in tumor tissue self-renewal and metastasis, no previously published biological or preclinical data could be identified to explain the propensity of *EGFR*-mutant NSCLC to develop BM and this remains to be explored [32]. Indeed, cancer invasiveness is stimulated by numerous mechanisms, including activating *EGFR* mutations [33]. Furthermore, a recent meta-analysis by Wang and Wang [34] exploring the correlation of *EGFR* status between primary tumors and metastases revealed that *EGFR* mutations should occur before metastasis, suggesting that *EGFR* mutations play a role in this process.

The main limitation of this study is its retrospective design. In addition, the population analyzed is relatively old since patients were diagnosed with advanced NSCLC from 2009 to 2011. However, this population was chosen to obtain mature OS data and to improve the homogeneity in terms of systemic therapies.

Future investigations are needed to elucidate the biological mechanisms underlying the association between *EGFR* mutation and higher rate of BM development in NSCLC patients. Furthermore, it will be important to assess how these results will impact treatment and follow-up of patients with *EGFR*-mutant NSCLC. Potential impact include an increase in the implementation of brain imaging evaluations with brain MRI (magnetic resonance imaging) or the use of new EGFR-TKIs with a better BBB penetration. A preclinical study recently revealed that osimertinib was a better option than gefitinib, rociletinib or afatinib for *EGFR*-mutant NSCLC and brain metastases, with a greater brain exposure and BBB penetration [35]. Finally, in this study, we focused on *EGFR* and *KRAS* mutations. However, other biomarkers may be associated with NSCLC BM incidence or prognosis. For example, *ALK* rearrangement has also been described as a risk factor for BM development [36]. For this reason, we plan to conduct a study of NSCLC BM biomarkers including whole exome data.

## 4. Experimental Section

### 4.1. Patients and Data Collection

Patients diagnosed at the Multidisciplinary Oncology and Therapeutic Innovations department (MOTI) of Aix Marseille University Hospital from January 2009 to December 2010 with metastatic non-squamous non-small cell lung cancer (ns-NSCLC) were included. In this study, inclusion criteria were the following: ns-NSCLC, stage IV at diagnosis, age above 18 years, and *EGFR* and *KRAS* mutations testing. Exclusion criteria were squamous cell carcinoma and/or no molecular testing. Patients with both *EGFR* and *KRAS* mutant tumors were also excluded this is an uncommon molecular profile that could not have been analyzed separately because of the small size (*n* = 2) of this population and would have induced statistical bias if they were analyzed twice with the other groups of patients with a single mutation.

Disease stage was assessed using the 7th IASCL (International Association for the Study of Lung Cancer) TNM classification [37]. Treatment efficacy was assessed using RECIST 1.1 (Response Evaluation Criteria in Solid Tumors) criteria [38]. Demographics (age, sex, ethnicity), clinical characteristics (ECOG performance status, smoking history, disease stage and metastases localization), biological characteristics (pathology, and *EGFR* and *KRAS* mutation status), treatment and survival outcomes were retrieved from the electronic patients’ records. BM was considered as synchronous if discovered at the time of NSCLC diagnosis and metachronous if discovered on later evaluation imaging.

This study was approved by a national ethics committee (CEPRO, Comité d’Evaluation des Protocoles de Recherche Observationnelle, reference number 2015-041), according to French law.

### 4.2. Molecular Testing

*EGFR* and *KRAS* mutations were analyzed for each patient from formalin-fixed, paraffin-embedded tissue, either from the primary tumor, lymph node or metastasis.

#### 4.2.1. Pre-analytical Stage

Serial 4 µm-thick sections prepared from paraffin-embedded tumor samples were dissected. Genomic DNA was extracted using NucleoSpin^®^ 96 DNA blood kit (Macherey-Nagel, Düren, Germany) according to the manufacturer’s protocol. DNA elution was carried out in 200 µL of bovine serum albumin at 0.5 mg/mL. DNA was stored at −20 °C until use.

#### 4.2.2. High Resolution Melting (HRM)

PCR amplification and HRM analysis from genomic DNA were carried out on LightCycler^®^ 480 (Roche Diagnostics, Meylan, France). Amplification consisted on 10 min at 95 °C, followed by 40 cycles of 10 s at 95 °C and 20 s at 65 and 50 °C for *EGFR* and *KRAS,* respectively, and 30 s at 72 °C for elongation. PCR was then carried out on LightCycler^®^ 480. Mutant cell lines, wild-type DNA (placenta), and PCR negative controls were included together with patient samples. Temperature ramping (from 60 to 95 °C, rising by 0.02 °C/s) and fluorescence acquisition settings were recommended by the manufacturer. HRM curves were normalized for each sample, and compared with wild-type genomic DNA using LightCycler^®^ 480 gene-scanning software [39].

### 4.3. Sequencing

Sanger sequencing was performed only for samples defined as “abnormal” after qPCR-HRM, using Big Dye Terminator v3.1 cycle sequencing kit (Life Technologies, Villebon-sur-Yvette, France) after DNA purification (ExoSap-IT^®^), on Evo75^®^ (Tecan, Männedorf, Switzerland). Sequences were analyzed on 3500 or 3130 Dx Genetic Analyser^®^ (Applied Biosystems, Villebon-sur-Yvette, France).

### 4.4. Treatment

Systemic treatment was decided after an MTB (Molecular Tumor Board) discussion on the basis of the ASCO (American Society of Clinical Oncology) [40] and ESMO (European Society of Medical Oncology) guidelines [41]. Local BM treatment was decided after Multidisciplinary Tumor Board discussion according to European and French guidelines [42,43].

### 4.5. Statistical Analysis

Statistical analysis was performed using IBM SPSS Statistics for Windows, Version 20.0 (IBM SPSS Inc., Chicago, IL, United States of America (USA)). Continuous variables were expressed as the mean ± standard deviation (SD) or as median with range (min, max), and categorical variables were reported as count and percentages. Comparisons of mean values between two groups were performed using Student’s *t*-test or Mann–Whitney *U* test. Comparisons of percentages were performed using Chi-Square test or Fisher’s exact test, as appropriate. Independent-sample *t*-tests and one-way analysis of variance were used to compare continuous variables between more than two groups. Time-to-event endpoints were estimated by the Kaplan–Meier method and compared using the log-rank test. OS was defined as the time from diagnosis to death from any cause, censored at the date of last follow-up. PFS was defined as the time from the beginning of treatment to documented progression or death, censored at the date of the last documented disease evaluation. Medians were reported with 95% confidence interval. Univariate and multivariate Cox proportional hazard regression models were used to estimate the HR. Multivariate analyses were performed when *p* < 0.2 or for clinically relevant parameters. For all the tests, a *p*-value <0.05 was considered significant.

## 5. Conclusions

This is the first study reporting the predictive role of *EGFR* and *KRAS* mutations on BM incidence, recurrence and patients’ outcomes in a Caucasian cohort of NSCLC patients. *EGFR* mutations are predictive for a higher incidence of BM while *KRAS* mutations are predictive for a lower rate of BM recurrence after local treatment and shorter survival. These results have to be validated in further studies and potentially. However, they already impact the routine management of NSCLC patients, highlignting the need for an increase in the implementation of brain imaging evaluations with brain MRI or the use of new EGFR-TKIs with a better BBB penetration.

## Figures and Tables

**Table 1:** Patient and disease characteristics associated with mutation status. WT: wild type; F: female; M: male; ECOG PS: ECOG performance status; *: significant *p*-values (*p* ≤ 0.05).

Characteristics	Total *n* (%)	*EGFR* Mutant *n* (%)	*KRAS* Mutant *n* (%)	*EGFR* and *KRAS* WT *n* (%)	*p*-Value
**Overall *n* (%)**	142 (100.00)	16 (11.27)	47 (33.10)	79 (55.63)	
**Patients’ Characteristics**					
Median age (years)	62 (31–88)	62 (45–87)	60 (37–76)	63 (31–88)	0.338
Sex F/M	48/94	7/9	16/31	25/54	0.46
Ethnicity					0.006 *
Caucasian	140 (98.59)	16 (100.00)	47 (100.00)	77 (97.47)	
Asian	2 (1.41)	0 (0.00)	0 (0.00)	2 (2.53)	
Smoking history					
Smoker	108 (76.06)	7 (43.75)	42 (89.36)	59 (74.68)	<0.001 *
Non-smoker	31 (21.83)	9 (56.25)	3 (6.38)	19 (24.05)	
ECOG PS					
0–1	123 (86.62)	14 (87.50)	37 (78.72)	72 (91.14)	0.131
≥2	19 (13.38)	2 (12.50)	10 (21.28)	7 (8.86)	
**Disease Characteristics**					
Pathology					
Adenocarcinoma	127 (89.44)	14 (87.50)	46 (97.87)	67 (84.81)	0.050 *
Other	15 (10.56)	2 (12.50)	1 (2.13)	12 (15.19)	
Number of metastases					
1	90 (63.38)	10 (62.50)	27 (57.45)	53 (67.09)	0.553
≥2	52 (36.62)	6 (37.50)	20 (42.55)	26 (32.91)	

**Table 2:** Association between mutation status and brain metastasis incidence and recurrence. WT: wild type; BM: brain metastases; *: significant *p*-value.

BM Characteristics	Total *n* (%)	*EGFR* Mutant *n* (%)	*KRAS* Mutant *n* (%)	*EGFR* and *KRAS* WT *n* (%)	*p*-Value
**BM Incidence**					
Brain Metastasis					0.031 *
Yes	81 (57.04)	14 (87.50)	26 (55.32)	41 (51.90)	
No	61 (42.96)	2 (12.50)	21 (44.68)	38 (48.10)	
Synchronous	52 (64.20)	8 (57.14)	18 (69.23)	26 (63.41)	0.741
Metachronous	29 (35.80)	6 (42.86)	8 (30.77)	15 (26.59)	
Brain Metastasis Related Death					0.528
Yes	31 (42.26)	7 (50.00)	11 (44.00)	13 (34.21)	
No	46 (59.74)	7 (50.00)	14 (56.00)	25 (65.79)	
**BM Recurrence**					
Recurrence					0.047 *
Yes *n* (%)	41 (50.62)	8 (57.14)	8 (30.77)	25 (60.98)	
No *n* (%)	40 (49.38)	6 (42.86)	18 (69.23)	16 (39.02)	
Time to Recurrence					1.000
≤12 months *n* (%)	29 (70.73)	6 (75.00)	6 (75.00)	17 (68.00)	
>12 months *n* (%)	12 (29.27)	2 (25.00)	2 (25.00)	8 (32.00)	

**Table 3:** Univariate and multivariate analyses of BM incidence and recurrence. OR: odds ratio; 95% CI: 95% confidence interval; BM: brain metastasis; age: <65 versus ≥65; sex female versus male; ethnicity: Caucasian versus non Caucasian; smoking status: smoker versus non-smoker; primitive tumor local treatment: surgery or radiation therapy versus no local treatment; Mutation status: *EGFR*-mutant versus *KRAS*-mutant versus *KRAS* and *EGFR* wild-type tumors; WT: wild-type; BM local treatment: any local treatment (whole brain radiation therapy, surgery or radiosurgery) versus no local treatment; number of metastases: one metastasis versus several metastases; NA: not applicable; *: significant *p*-value.

Characteristics	Univariate Analyses			Multivariate Analyses		
	OR	95% CI	*p*-Value	OR	95% CI	*p*-Value
**BM Incidence**						
Age	0.996	0.965–1.027	0.799	0.998	0.965–1.032	0.897
Sex	1.193	0.592–2.404	0.621	0.988	0.440–2.219	0.977
ECOG PS	1.041	0.391–2.769	0.936	NA	NA	NA
Smoking status	0.767	0.345–1.706	0.515	0.489	0.178–1.339	0.164
Primary tumor local treatment	1.384	0.541–3.545	0.498	NA	NA	NA
Mutation status			0.060			0.031 *
*EGFR* mutant versus *EGFR* WT	6.488	1.383–30.443	0.018 *	8.745	1.743–43.881	0.008 *
*KRAS* mutant versus *KRAS* WT	1.148	0.556–2.369	0.710	1.082	0.505–2.318	0.840
**BM Recurrence after Local Treatment**						
Age	0.987	0.944–1.032	0.562	0.975	0.929–1.023	0.296
Sex	1.037	0.408–2.636	0.939	0.983	0.322–3.000	0.976
ECOG PS	0.510	0.137–1.898	0.315	NA	NA	NA
Smoking status	0.939	0.314–2.810	0.911	0.626	0.153–2.565	0.515
**Mutation status**			0.054			0.029 *
*EGFR* mutant versus *EGFR* WT	0.853	0.249–2.921	0.801	0.970	0.245–3.845	0.966
*KRAS* mutant versus *KRAS* WT	0.284	0.100–0.807	0.018 *	0.234	0.078–0.699	0.009 *
Synchronous or metachronous BM	1.786	0.712–4.480	0.216	NA	NA	NA
BM local treatment	0.709	0.236–2.133	0.541	NA	NA	NA
Number of metastases	0.962	0.390–2.370	0.932	NA	NA	NA

**Table 4:** Association between mutation status and survival. OS and PFS and mutation status in all patients and in patients with BM segregated into two groups. WT: wild type; PFS: progression free survival; OS: overall survival; SRS: stereotactic radio-surgery; WBRT; whole brain radiation therapy; *: significant *p*-value.

Population	*EGFR* Mutant	*KRAS* Mutant	*EGFR* and *KRAS* WT	*p*-Value
PFS and OS				
**All patients *n* (%)** Median OS (months)	16 (11.27) 22 (4.36–39.64)	47 (33.10) 9 (7.12–10.88)	79 (55.63) 18 (12.30–23.70)	0.196
**Patients with BM *n* (%)**	14 (17.28)	26 (32.10)	41 (50.62)	
Surgery and/or SRS				
Median PFS (months) Median OS (months)	7 (6.12–7.88) 30 (4.24–55.77)	9 (5.67–12.33) 22 (7.58–36.42)	8 (5.08–9.16) 25 (20.44–29.56)	0.227 0.822
WBRT only				
Median PFS (months) Median OS (months)	11 (0.00–23.88) 28 (0.00–73.09)	9 (1.30–16.70) 7 (1.87–12.13)	6 (5.08–6.92) 12 (5.36–18.65)	0.272 0.208
No local treatment				
Median PFS (months) Median OS (months)	10 (8.40–11.60) 19 (1.85–36.15)	5 (1.80–8.20) 5 (0.00–12.85)	6 (3.23–8.77) 14 (12.22–14.78)	0.229 0.010 *

**Table 5:** Univariate and multivariate analyses of overall survival in patients with untreated brain metastasis. OR: odds ratio; 95% CI: 95% confidence interval; BM: brain metastasis; age: <65 versus ≥65; sex female versus male; ethnicity: Caucasian versus non-Caucasian; smoking status: smoker versus nonsmoker; primary tumor local treatment: surgery or radiation therapy versus no local treatment; Mutation status: *EGFR* mutant versus *KRAS* mutant versus *KRAS* and *EGFR* wild type tumors; WT: wild-type; number of metastases: one metastasis versus several metastases; *: significant *p*-value.

Characteristics	Univariate Analyses			Multivariate Analyses		
	OR	95% CI	*p*-Value	OR	95% CI	*p*-Value
**OS of Patients with Untreated BM**						
Age	1.018	0.968–1.071	0.484	NA	NA	NA
Sex	1.41	0.310–6.044	0.657	NA	NA	NA
ECOG PS	8.366	1.643–42.606	0.011 *	8.768	0.493–155.954	0.139
Smoking status	0.951	0.203–4.449	0.949	NA	NA	NA
Mutation status			0.010 *			0.698
*EGFR* mutant versus *EGFR* WT	0.536	0.136–2.115	0.374	0.589	0.134–2.581	0.482
*KRAS* mutant versus *KRAS* WT	7.130	1.240–41.012	0.028 *	0.356	0.015–8.224	0.519
Synchronous or metachronous BM	1.017	0.339–3.047	0.976	NA	NA	NA
Number of metastases	11.548	2236–59.640	0.003 *	6.669	1.022–43.541	0.047 *

**Table 6:** Case series assessing the association of molecular profile with NSCLC BM. NSCLC = non-small cell lung cancer, ADC = adenocarcinoma, BM = brain metastases, NA = not applicable.

References	Patients Number	Ethnicity	Pathology	NSCLC Stage	Results
[22]	314	Asian	ADC	All	Association between *EGFR* mutation and BM incidence
[23]	401	Asian	NSCLC	All	Association between *EGFR* mutation and BM incidence
[24]	259	Asian	NSCLC	Advanced	Association between *EGFR* mutation and synchronous BM and longer median BM-OS
[25]	481	Caucasian	NSCLC	Early	*EGFR* mutations predict BM, *KRAS* mutations predict pleuro-pericardial metastases
[26]	189	Caucasian	NSCLC	All	No association between *EGFR* and *KRAS* mutation status and BM incidence
[27]	118	NA	NSCLC	IV	No association between *EGFR* mutation status and BM incidence
[28]	100	Asian	EGFR-mutant ADC	All	Association between *EGFR* exon 19 deletion and BM incidence
[29]	55	Caucasian	EGFR-mutant NSCLC	All	Association between *EGFR* exon 19 deletion and BM incidence
[30]	236	NA	NSCLC	IV (BM)	Patients with *EGFR* mutant BM had improved survival
[31]	106	Asian	NSCLC	IV (BM)	Exon 19 deletion is an independent prognostic factor in BM from NSCLC

## Supplementary Materials

Supplementary materials can be found at www.mdpi.com/1422-0067/17/12/2132/s1.
