# Supplementary material for: EGFR and KRAS Mutations Predict the Incidence and Outcome of Brain Metastases in Non-Small Cell Lung Cancer
Source: Int J Mol Sci. 2016 Dec 18;17(12):2132. doi: 10.3390/ijms17122132 (PMC5187932; doi:10.3390/ijms17122132)
Supplement: Supplementary file 1 [file ijms-17-02132-s001.pdf]

Supplementary Materials: *EGFR* and *KRAS* Mutations Predict the Incidence and Outcome of Brain Metastases in Non-small Cell Lung Cancer

Pascale Tomasini, Cindy Serdjebi, Nataliya Khobta, Philippe Metellus, L'Houcine Ouafik, Isabelle Nanni, Laurent Greillier, Anderson Loundou, Frederic Fina, Celine Mascaux and Fabrice Barlesi

Table S1. *EGFR* and *KRAS* mutations identified. *EGFR* mutations were classified according to their location on DNA sequence (exon), whereas *KRAS* mutations were stratified according to their location on the mRNA (codon).

EGFR Mutations			*KRAS* Mutations		
Base change	Amino acid change	Patients *n* (%)	Base change	Amino-acid change	Patients *n* (%)
Subjects		16 (11.27)	Subjects		47 (33.10)
Exon18			Codon 12		
c.2104G>C	Ala702Pro	1 (6.25)	c.34G>T	Gly12Cys	27 (57.45)
Exon 19			c.34G>A	Gly12Ser	3 (6.38)
c.2235_2249del	Glu746_Ala750del	8 (50.00)	c.35G>C	Gly12Ala	2 (4.26)
c.2235_2255 delinsAAT	Glu746_Ser752delinsIle	1 (6.25)	c.35G>A	Gly12Asp	7 (14.89)
c.2237_2251del	Glu746_Thr751delInsAla	1 (6.25)	c.35G>T	Gly12Val	5 (10.64)
c.2237_2255delinsT	Glu746_Ser752delinsVal	1 (6.25)	Codon 13		
Exon 21			c.37G>T	Gly13Cys	1 (2.13)
c.2573T>G	Leu858Arg	3 (18.75)	c.38G>A	Gly13Asp	2 (4.26)
c.2582T>A	Leu861Gln	1 (6.25)			
